# Prognostic significance and immune correlates of CD73 expression in renal cell carcinoma

**DOI:** 10.1136/jitc-2020-001467

**Published:** 2020-11-11

**Authors:** Abhishek Tripathi, Edwin Lin, Wanling Xie, Abdallah Flaifel, John A Steinharter, Emily N Stern Gatof, Gabrielle Bouchard, Justin H Fleischer, Nieves Martinez-Chanza, Connor Gray, Charlene Mantia, Linda Thompson, Xiao X Wei, Marios Giannakis, Bradley A McGregor, Toni K Choueiri, Neeraj Agarwal, David F McDermott, Sabina Signoretti, Lauren C Harshman

**Affiliations:** 1 University of Oklahoma Health Sciences Center, Stephenson Cancer Center, Oklahoma City, Oklahoma, USA; 2 Lank Center for Genitourinary Oncology, Dana-Farber Cancer Institute, Boston, Massachusetts, USA; 3 University of Utah, Huntsman Cancer Institute, Salt Lake City, Utah, USA; 4 Department of Data Sciences, Dana Farber Cancer Institute, Boston, Massachusetts, USA; 5 Brigham and Women's Hospital, Boston, Massachusetts, USA; 6 Beth Israel Deaconess Medical Center, Boston, Massachusetts, USA; 7 Oklahoma Medical Research Foundation, Oklahoma City, Oklahoma, USA; 8 Dana-Farber Cancer Institute, Boston, Massachusetts, USA

**Keywords:** adenosine, kidney neoplasms, immunotherapy, biomarkers, tumor, gene expression profiling

## Abstract

**Background:**

CD73–adenosine signaling in the tumor microenvironment is immunosuppressive and may be associated with aggressive renal cell carcinoma (RCC). We investigated the prognostic significance of CD73 protein expression in RCC leveraging nephrectomy samples. We also performed a complementary analysis using The Cancer Genome Atlas (TCGA) dataset to evaluate the correlation of CD73 (ecto-5′-nucleotidase (*NT5E*), CD39 (ectonucleoside triphosphate diphosphohydrolase 1 (*ENTPD1*)) and A2 adenosine receptor (A2AR; *ADORA2A*) transcript levels with markers of angiogenesis and antitumor immune response.

**Methods:**

Patients with RCC with available archived nephrectomy samples were eligible for inclusion. Tumor CD73 protein expression was assessed by immunohistochemistry and quantified using a combined score (CS: % positive cells×intensity). Samples were categorized as CD73_negative_ (CS=0), CD73_low_ or CD73_high_ (< and ≥median CS, respectively). Multivariable Cox regression analysis compared disease-free survival (DFS) and overall survival (OS) between CD73 expression groups. In the TCGA dataset, samples were categorized as low, intermediate and high *NT5E*, *ENTPD1* and *ADORA2A* gene expression groups. Gene expression signatures for infiltrating immune cells, angiogenesis, myeloid inflammation, and effector T-cell response were compared between *NT5E*, *ENTPD1* and *ADORA2A* expression groups.

**Results:**

Among the 138 patients eligible for inclusion, ‘any’ CD73 expression was observed in 30% of primary tumor samples. High CD73 expression was more frequent in patients with M1 RCC (29% vs 12% M0), grade 4 tumors (27% vs 13% grade 3 vs 15% grades 1 and 2), advanced T-stage (≥T3: 22% vs T2: 19% vs T1: 12%) and tumors with sarcomatoid histology (50% vs 12%). In the M0 cohort (n=107), patients with CD73_high_ tumor expression had significantly worse 5-year DFS (42%) and 10-year OS (22%) compared with those in the CD73_negative_ group (DFS: 75%, adjusted HR: 2.7, 95% CI 1.3 to 5.9, p=0.01; OS: 64%, adjusted HR: 2.6, 95% CI 1.2 to 5.8, p=0.02) independent of tumor stage and grade. In the TCGA analysis, high *NT5E* expression was associated with significantly worse 5-year OS (p=0.008). *NT5E* and *ENTPD1* expression correlated with higher regulatory T cell (Treg) signature, while *ADORA2A* expression was associated with increased Treg and angiogenesis signatures.

**Conclusions:**

High CD73 expression portends significantly worse survival outcomes independent of stage and grade. Our findings provide compelling support for targeting the immunosuppressive and proangiogenic CD73–adenosine pathway in RCC.

## Introduction

Immune checkpoint inhibitors targeting the programmed cell death-1 (PD-1) and cytotoxic T-lymphocyte-associated antigen-4 (CTLA-4) pathways have significantly advanced the treatment of metastatic renal cell carcinoma (mRCC). Recent studies combining PD-1/L1 inhibitors with either the vascular endothelial growth factor (VEGF) receptor tyrosine kinase inhibitor axitinib or the anti-CTLA-4 antibody ipilimumab have demonstrated improved objective response rates (ORRs), progression-free survival (PFS) and overall survival (OS) compared with sunitinib.^1–4^ Given these results, most patients with clear cell renal cell carcinoma (ccRCC) will receive a PD-1/L1 inhibitor-based regimen either in combination with axitinib or, if intermediate or poor-risk disease, ipilimumab, as first-line therapy. Although immune checkpoint blockade represents a significant therapeutic advance, approximately 20% of patients are primary treatment-refractory to these agents, and the majority who experience clinical benefit eventually progress as evidenced by a median PFS of 12–14 months on any of these regimens.^1–4^ The lack of response in a significant proportion of patients and eventual disease progression in most patients suggests the existence of multiple non-redundant mechanisms of de novo or acquired resistance to immune checkpoint inhibition in the tumor microenvironment.

CD73 (ecto-5′-nucleotidase (*NT5E*)) and CD39 (ectonucleoside triphosphate diphosphohydrolase 1 (*ENTPD1*)) mediate sequential dephosphorylation of extracellular ATP to adenosine with CD73 catalyzing the rate-limiting step.^5^ A hypoxic tumor microenvironment significantly increases CD73 expression on tumor cells and tumor-inﬁltrating immune cells through hypoxia-inducible factor (HIF)-1α.^6–8^ Adenosine generated by CD73 and CD39 binds to and activates G protein-coupled A1, A2A, A2B, and A3 receptors.^9^ In preclinical models, increased adenosine signaling attenuates the antitumor immune response through the proliferation of regulatory T cells (Tregs) and myeloid-derived suppressor cells (MDSCs) and differentiation of tumor-associated macrophages into the immunosuppressive M2 phenotype.^10–13^ Adenosine signaling may also stimulate angiogenesis through increased vasodilation, release of proangiogenic factors such as VEGF, and recruitment of endothelial progenitor cells in the tumor microenvironment.^14–16^

Previously, our group found that CD73 was more frequently expressed in mRCC specimens compared with primary tumors.^17^ As CD73 mediates the rate-limiting step in the generation of immunosuppressive adenosine, we hypothesized that higher CD73 expression correlates with more aggressive disease in renal cell carcinoma (RCC). Employing an institutional dataset of RCC samples, we investigated the prognostic significance of CD73 expression in localized RCC. Using gene expression data from The Cancer Genome Atlas (TCGA) Kidney Renal Clear Cell Carcinoma (KIRC) dataset, we also evaluated the correlation of mRNA expression of CD73 (*NT5E*), CD39 (*ENTPD1*) and A2AR (*ADORA2A*) with gene expression signatures reflecting angiogenesis, myeloid inflammation and effector T-cell response (T_eff_) and infiltrating immune cell subsets.

## Methods

### Institutional dataset

The study used previously established RCC tissue microarrays (TMAs) which included primary tumor samples from patients with localized or de novo mRCC (T1–4, N0–1, M0–1), who underwent nephrectomy at Dana-Farber Cancer Institute/Brigham and Women’s Hospital and Beth Israel Deaconess Medical Center between January 2002 and May 2006. Immunohistochemistry (IHC) for CD73 was performed on the formalin-fixed paraffin-embedded tumor tissue from the TMAs. Rehydrated tissue sections were boiled in citrate buffer pH 6.0 (Life Technologies, Frederick, Maryland, USA) with a pressure cooker (Biocare Medical, Pacheco, California, USA) for 30 s at 125°C. After cooling down at room temperature (RT), tissue sections were successively incubated with a peroxidase block (Dual Endogenous Enzyme Block, Agilent) and a protein block (Serum Free Block, Agilent) for 10 min each. Sections were next incubated for 1 hour at RT with the rabbit monoclonal anti-CD73 antibody (clone D7F9A, 1:25; Cell Signaling Technology) diluted in antibody diluent with background reducing components (Agilent). Tissue sections were then incubated for 1 hour at RT with a polyclonal mouse anti-rabbit antibody (Agilent, 1:750) diluted in antibody diluent with background reducing components (Agilent) followed by incubation for 30 min at RT with EnVision anti-mouse horseradish peroxidase (HRP)-conjugated antibody (Agilent). HRP visualization was performed by applying 3,3-diaminobenzidine+substrate (Agilent) for 1 min and 30 s. Between steps, tissue sections were washed for 5 min in washing buffer (0.1 mM Tris, pH 7.4+0.05% Tween 20). Nuclei were counterstained with hematoxylin.

von Hippel-Lindau deficient and proficient University of Michigan-Renal Carcinoma-2 cells were used as positive and negative controls, respectively. Percentage of cell staining positive for CD73 and the intensity of staining (1+, 2+ or 3+) were assessed manually by one observer (SS). CD73 expression for each sample was quantified using a combined score (CS), which was calculated by multiplying the intensity of staining (1+, 2+, or 3+) with the percentage of tumor cells staining positive. For patients with multiple tumor samples with evaluable CD73 expression, the mean CS of all available samples was calculated. CD73 positivity was defined as any CD73 expression on tumor cells irrespective of percentage of cells or the intensity of staining. Patients with CD73 positivity were categorized into CD73_low_ and CD73_high_ subgroups. Patients with CS less than the median CS of all CD73-positive patients were designated as CD73_low_. Conversely, those with expression equal to or higher than the median CS were characterized as CD73_high_. Clinical and pathological characteristics such as tumor stage, Fuhrman grade, presence or absence of sarcomatoid features, along with outcomes data, were cataloged through retrospective chart review by investigators blinded to the CD73 expression data.

### TCGA dataset

RNA-seq data for 538 ccRCC samples derived from primary tumors were obtained from the TCGA-KIRC dataset.^18^ Gene expression of CD73 (*NT5E*), CD39 (*ENTPD1*) and A2AR (*ADORA2A*) was quantified by HT-seq and measured in upper-quartile normalized fragments per kilobase million.^19^ Patients were split into low (≤−1 SD from the overall mean), intermediate (−1 to 1 SD from the mean), and high (≥1 SD from the mean) *NT5E*, *ENTPD1* and *ADORA2A* expression groups. Averaged log2-transformed expression of previously validated genes was used to calculate angiogenesis (*VEGFA*, *KDR*, *ESM1*, *PECAM1*, *ANGPTL4* and *CD34*), myeloid inflammation (*IL-6*, *CXCL1*, *CXCL2*, *CXCL3*, *CXCL8* and *PTGS2*), and T_eff_ (*CD8A*, *EOMES*, *PRF1, IFNG* and *CD274*) gene expression signatures.^20^ Gene signatures for tumor-infiltrating immune cells such as B cells, CD4^+^ T cells, CD8^+^ T cells, neutrophils, NK cells, macrophages, and dendritic cells were calculated based on averaged log-transformed expression levels of previously validated immune metagenes.^21^

### Statistical analysis

Baseline patient characteristics were summarized using descriptive statistics. The distribution of CD73 expression in the primary tumor (CD73_negative_ vs CD73_low_ or CD73_high_) by baseline clinical and pathologic characteristics was compared using the Cochran-Armitage Trend test (for variables with two categories) and Jonckheere-Terpstra test (for variables three or more ordered categories). The primary survival analysis focused on patients with localized RCC for which disease-free survival (DFS) and OS were primary outcome measures. DFS was defined as the time from nephrectomy to disease recurrence or death from any cause. OS was defined as the time from nephrectomy to death from any cause. DFS and OS were censored at the date of the last follow-up. An exploratory analysis evaluated outcomes between CD73 expression groups in patients with de novo mRCC. For this analysis, OS was the primary outcome measure and defined as the time from cytoreductive nephrectomy to death from any cause. Distribution of DFS and OS were estimated using the Kaplan Meier method. Multivariable Cox regression analysis was used to estimate the association of CD73 expression with DFS and OS adjusted for known prognostic factors tumor grade (grade ≥3 vs<3) and the American Joint Committee on Cancer (AJCC) disease stage (III/IV vs I/II). All statistical tests were two-sided and statistical significance was considered at p<0.05.

In the TCGA dataset, the Mann-Whitney U test was used to compare AJCC disease stage and gene expression signature scores between low, intermediate, and high *NT5E*, *ENTPD1* and *ADORA2A* expression groups. Bonferroni correction was used to control false discovery rate to ⍺<0.05. Significant differences were defined by a fold change >4 and adjusted p<0.05. OS was compared between low, intermediate, and high *NT5E* and *ADORA2A* groups using the Log rank test stratified by AJCC stage. All bioinformatic analyzes and figures using TCGA data were produced using Pandas, NumPy, SciPy, and Matplotlib software packages in a Python 3.6 environment.

## Results

### CD73 expression and outcomes in the Dana-Farber/Harvard Cancer Center institutional dataset

We interrogated TMAs consisting of tumor samples from 199 patients with localized or de novo mRCC. For our analysis, patients were excluded if pathologic diagnosis was not RCC (n=17), they had unevaluable CD73 expression (n=13), or there was inadequate linked clinical and pathological data (n=19) (online supplemental figure 1). In order to minimize potential heterogeneity induced by higher CD73 expression in metastatic samples compared with primary tumors,^17^ we excluded patients who only had samples from metastatic sites (n=12). Of the 138 eligible patients with primary tumor samples, 107 patients had localized RCC, and 31 patients had de novo mRCC. ccRCC was the most common histologic subtype (75%, n=103; table 1) with 24% non-clear cell disease (papillary, n=16; chromophobe, n=14). High nuclear grade (≥3) and sarcomatoid component was present in 43% (n=60) and 7% (n=10) respectively. The majority (n=88, 64%) had pT1-T2 tumors while 33% (n=46) were ≥pT3 and 10% had nodal involvement (n=14). Locally advanced disease defined as AJCC stage III or IV was present in 44% (n=60) of patients.

10.1136/jitc-2020-001467.supp1 Supplementary data

**Table 1:** Baseline characteristics of patients (N=138)

	Localized RCC (N=107)		De novo mRCC (N=31)		Total (N=138)	
	n	%	n	%	n	%
Gender						
Female	43	40	14	45	57	41
Male	64	60	17	55	81	59
Histology						
ccRCC	78	73	25	81	103	75
nccRCC	28	26	5	16	33	24
Chromophobe	14	13	.	.	14	10
Papillary not subtyped	6	6	1	3	7	5
Papillary type 1	4	4	1	3	5	4
Papillary type 2	3	3	1	3	4	3
RCC unclassified	1	1	2	7	3	2
Unknown	1	1	1	3	2	1
Pathological T stage						
T1	58	54	2	6	60	44
T2	21	20	7	23	28	20
T3	21	20	21	68	42	30
T4	3	3	1	3	4	3
Unknown	4	4	.	.	4	3
Pathological N stage						
N0	33	31	11	36	44	32
N1	8	8	6	19	14	10
Nx	62	58	14	45	76	55
Unknown	4	4	.	.	4	3
AJCC stage						
I	57	53	.	.	57	41
II	19	18	.	.	19	14
III (T3 or N1)	26	24	.	.	26	19
IV (T4 or M1)	3	3	31	100	34	25
Unknown	2	2	.	.	2	1
Fuhrman grade						
G1	7	7	.	.	7	5
G2	59	55	9	29	68	49
G3	26	24	12	39	38	28
G4	13	12	9	29	22	16
Unknown	2	2	1	3	3	2
Sarcomatoid features						
No	100	94	24	77	124	90
Yes	4	4	6	19	10	7
Unknown	3	3	1	3	4	3
Adjuvant therapy						
No	103	96	31	100	134	97
Yes	3	3	.	.	3	2
Unknown	1	1	.	.	1	1

AJCC, American Joint Committee on Cancer; ccRCC, clear cell renal cell carcinoma; mRCC, metastatic renal cell carcinoma; nccRCC, non-clear cell renal cell carcinoma; RCC, renal cell carcinoma.

**Figure 1:**
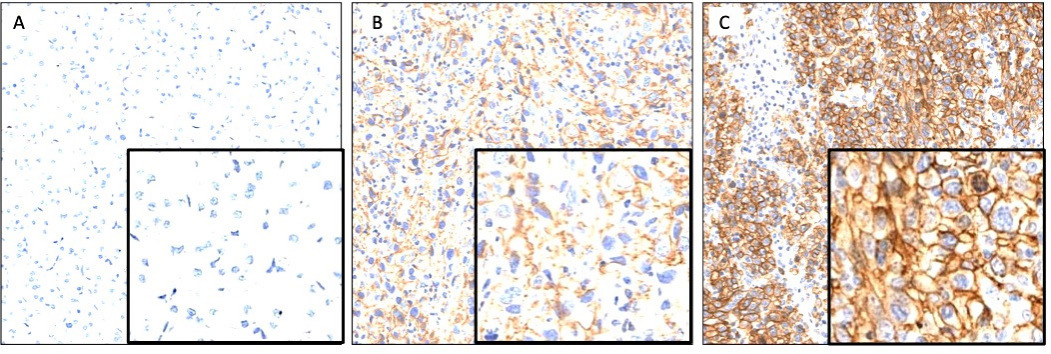
Representative images demonstrating membraneous CD73 expression on tumor cells in the CD73_negative_ (A), CD73_low_ (B) and CD73_high_ (C) groups.

**Figure 2:**
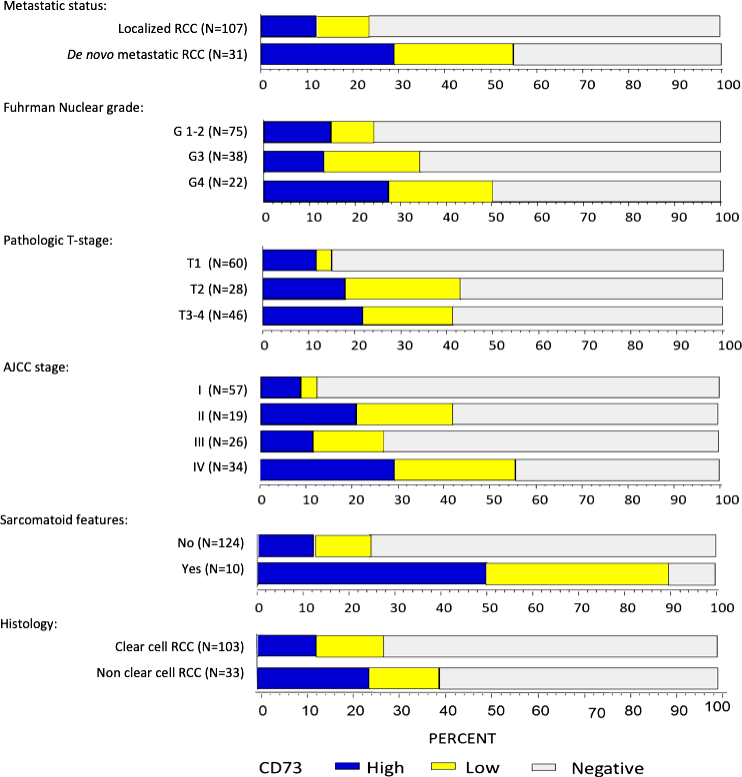
Distribution of CD73 expression (negative, low and high) by baseline clinical and pathological characteristics. AJCC, American Joint Committee on Cancer; RCC, renal cell carcinoma.

In the overall cohort (n=138), any CD73 expression (CS >0) was seen in 30% (n=42, table 2) of samples with a median CS of 11.8 (range 0.1–210, figure 1). In patients with ccRCC, CD73 positivity was seen in 27% of tumors (median CS: 6.3, range 0.1–125) and 13% had high CD73 expression. In patients with non-clear cell histology, CD73 positivity was observed in 39% with 24% being CD73_high_. Compared with patients with localized (M0) RCC, those with de novo mRCC were more likely to be CD73 positive (55% vs 23%; p=0.002) and have high CD73 expression (29% vs 12%, figure 2, p=0.002). Similarly, high CD73 expression was more frequent in grade four tumors (27%) compared with grade 3 (13%) or grade 1–2 (15%, p_trend_=0.035, figure 2), pathologic stage ≥T3 (22%) compared with T2 (19%) and T1 (12%; p_trend_=0.004) and locally advanced disease stage (AJCC stage, IV: 29% vs III: 12% vs II: 21% vs I: 9%; p_trend_ <0.0001). In tumors with sarcomatoid features (n=10), 90% (n=9) had any CD73 expression with 50% (n=5) being CD73_high_. In contrast, in tumors without sarcomatoid features, only 24% (n=30) demonstrated any CD73 expression and only 12% (n=15) were CD73_high_ (p<0.001).

**Table 2:** Associations of CD73 expression with baseline characteristics of patients (N=138)

Baseline characteristics (N)	CD73 expression						P value*
	Positive (N=42)				Negative (N=96)		
	High (n=22)		Low (n=20)				
	n	%	n	%	n	%	
Metastatic status at presentation							0.002
Localized RCC (n=107)	13	12	12	11	82	77	
De novo mRCC (n=31)	9	29	8	26	14	45	
Histology							0.11†
ccRCC (n=103)	13	13	15	15	75	73	
nccRCC (n=33)	8	24	5	15	20	61	
Papillary (n=16)	6	38	5	31	5	31	
Chromophobe (n=14)	2	14	.	.	12	86	
RCC unclassified (n=3)	.	.	.	.	3	100	
Unknown (n=2)	1	50			1	50	
Sarcomatoid features							<0.0001
No (n=124)	15	12	15	12	94	76	
Yes (n=10)	5	50	4	40	1	10	
Unknown (n=4)	2	50	1	25	1	25	
Fuhrman nuclear grade							0.035
G1-2 (n=75)	11	15	7	9	57	76	
G3 (n=38)	5	13	8	21	25	66	
G4 (n=22)	6	27	5	23	11	50	
Unknown (n=3)					3	100	
Pathological T stage							0.004
T1 (n=60)	7	12	2	3	51	85	
T2 (n=28)	5	19	7	25	16	57	
T3-4 (n=46)	10	22	9	20	27	59	
Unknown (n=4)			2	50	2	50	
AJCC stage							<0.0001
I (n=57)	5	9	2	4	50	88	
II (n=19)	4	21	4	21	11	58	
III (n=26)	3	12	4	15	19	73	
IV (n=34)	10	29	9	27	15	44	
Unknown (n=2)			1	50	1	50	

*Cochran-Armitage Trend test (for variables with two categories) and Jonckheere-Terpstra test (for variables with ≥3 ordered categories); unknown group was excluded from the comparison.

†P value is for comparison of ccRCC versus nccRCC.

AJCC, American Joint Committee on Cancer; ccRCC, clear cell renal cell carcinoma; mRCC, metastatic renal cell carcinoma; nccRCC, non-clear cell renal cell carcinoma; RCC, renal cell carcinoma.

**Figure 3:**
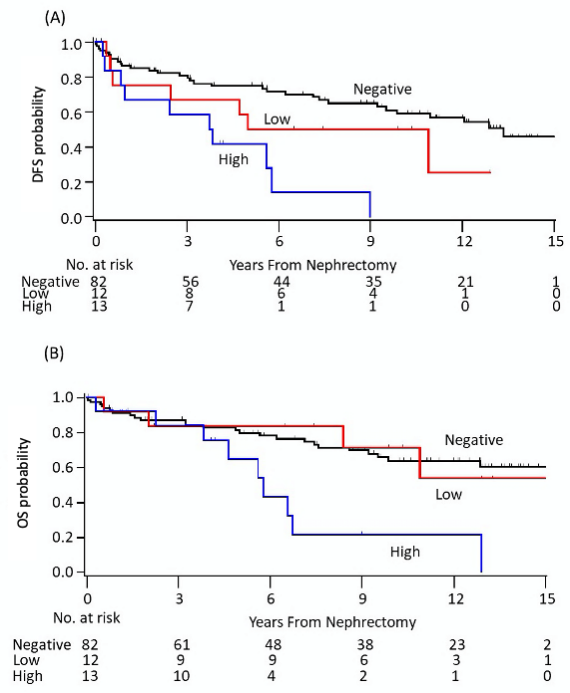
Kaplan-Meier plots of (A) DFS and (B) OS according to CD73 expression in patients with localized renal cell carcinoma. DFS, disease-free survival; OS, overall survival.

Median follow-up was 10 years (range <0.1–15.5 years) for patients with localized RCC (n=107). DFS at 5 years for patients in the CD73_negative_, CD73_low_ and CD73_high_ groups was 75%, 50% and 42%, respectively (figure 3A). On multivariable analysis adjusting for Fuhrman grade (graded 1–2 vs grades 3–4) and AJCC stage (stage I/II vs III/IV), high CD73 expression was associated with a significantly worse DFS (table 3; adjusted HR: 2.72, 95% CI 1.27 to 5.85, p=0.01). Similarly, the CD73_high_ group experienced significantly worse 10-year OS compared with the CD73_negative_ group (22% vs 64%, adjusted HR: 2.59, 95% CI 1.15 to 5.84, p=0.021; figure 3B). The survival outcomes were not significantly different between the CD73_negative_ and CD73_low_ groups (DFS HR: 1.22, 95% CI 0.50 to 2.98; OS HR: 0.78, 95% CI 0.27 to 2.30). Additional analysis combining the CD73 negative and low groups was performed and the significant differences in DFS and OS remained. The adjusted HR for DFS was 2.89 (95%CI: 1.37 to 6.07, p=0.005) and for OS was 2.86 (95% CI 1.30 to 6.29, p=0.009) in favor of no or low expression (CD73_high_ vs CD73_negative+low_) (table 3). In an exploratory analysis evaluating OS in patients with de novo mRCC (n=31), there was no statistically significant difference in 2-year OS between patients in the CD73_negative_ (43%), CD73_low_ (50%) and CD73_high_ groups (52%, online supplemental figure 2; p=0.52).

**Table 3:** Multivariable analysis comparing DFS and OS between CD73 expression groups in patients with localized RCC

		DFS				OS			
CD73 expression groups	N	Event (n)	5-year DFS, % (95% CI)	Adjusted HR (95% CI)*	Adjusted P value	Event (n)	10 year OS, % (95% CI)	Adjusted HR (95% CI)*	Adjusted P value
CD73_negative_	82	32	75 (63 to 83)	Reference		25	64 (51 to 74)	Reference	
CD73_low_	12	7	50 (21 to 74)	1.22 (0.50 to 2.98)	0.659	5	71 (34 to 90)	0.78 (0.27 to 2.30)	0.655
CD73_high_	13	10	42 (15 to 67)	**2.72 (1.27 to 5.85**)	**0.010**	9	22 (4 to 50)	**2.59 (1.15 to 5.84**)	**0.021**
CD73_high_ vs CD73_low+negative_				**2.89 (1.37 to 6.07**)	**0.005**			**2.86 (1.30 to 6.29**)	**0.009**

Results in bold indicate statistically significant results

*Adjusted for Fuhrman nuclear grade (G1–2 vs G3–4) and AJCC stage (stage I/II vs III/IV). Multivariable models excluded four patients with unknown grade or stage.

AJCC, American Joint Committee on Cancer; DFS, disease-free survival; OS, overall survival; RCC, renal cell carcinoma.

### Clinical and genomic correlates of *NT5E*, *ENTPD1* and *ADORA2A* gene expression in the TCGA dataset

**Figure 4:**
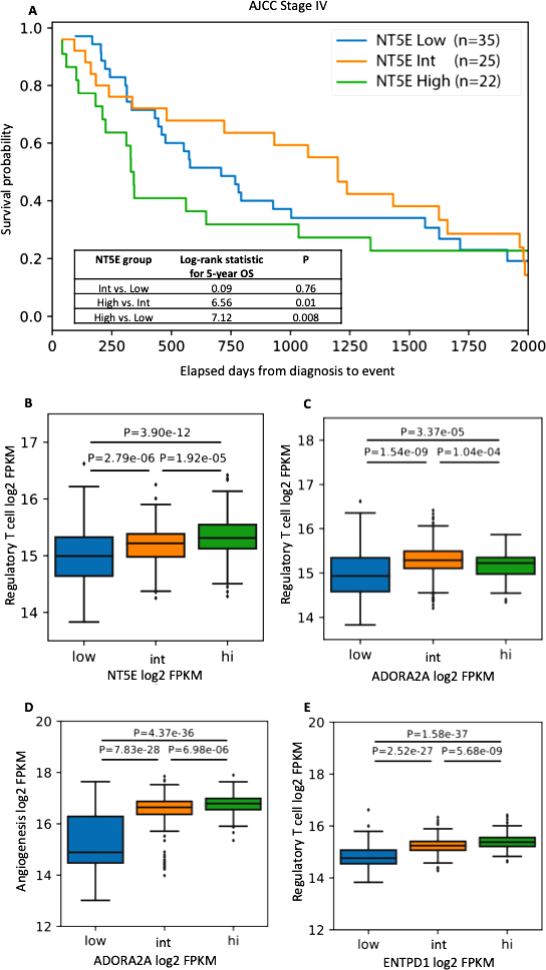
Genomic and clinical correlates of CD73 (*NT5E*), A2aR (*ADORA2A*) and CD39 (*ENTPD1*) gene expression in the TCGA cohort. AJCC, American Joint Committee on Cancer; FPKM, fragments per kilobase million; Int, intermediate; OS, overall survival.

In the complementary analysis using TCGA RNA-seq data, high *NT5E* expression was associated with significantly lower 5-year OS compared with intermediate or low *NT5E* expression (figure 4A) in AJCC stage IV tumors. No significant differences in OS were observed between *NT5E* expression groups in patients with AJCC stage I–III disease (online supplemental figure 3A-C). *NT5E* expression was similar across AJCC stages (online supplemental figure 3D). Similarly, there was no significant correlation of *ADORA2A* expression with OS or AJCC disease stage (online supplemental figure 4). Treg gene expression signature was positively correlated with *NT5E*, *ENTPD1* and *ADORA2A* expression (figure 4). The angiogenesis gene expression signature positively correlated with *ADORA2A* (figure 4D) but not *NT5E* or *ENTPD1* expression (online supplemental tables 1-3). T_eff_, myeloid inflammation, and other immune cell signatures (eg, CD8^+^ T cells, B cells, neutrophils, and macrophages) were not significantly different between the low intermediate and high *NT5E*, *ENTPD1* or *ADORA2A* expression groups.

## Discussion

In this biomarker-based analysis, CD73 protein expression was present in 30% of all RCC nephrectomy tumor specimens. Higher expression correlated with more aggressive disease as defined by higher Fuhrman nuclear grade, advanced stage at diagnosis, presence of sarcomatoid histology, and in patients with localized RCC, significantly worse DFS and OS. In a complementary analysis using the TCGA gene expression dataset, high *NT5E* (CD73) gene expression was associated with significantly worse OS in AJCC stage IV tumors. *NT5E* and *ENTPD1* expression correlated with increased expression of immunosuppressive Treg markers, while A2AR (*ADORA2A*) expression was associated with significantly higher angiogenesis and Treg gene expression signatures.

There is a sparse but growing literature on the prevalence and clinical significance of CD73 expression in RCC. Yu *et al* analyzed CD73 expression by IHC in patients with ccRCC, the majority of whom had localized (T1–4, N0, M0) disease.^22^ Nearly half of tumors (48%) expressed CD73, which was associated with high tumor grade and T stage. In our prior work in RCC, we observed CD73 expression in 19% of primary tumors and that expression was significantly more common in metastatic tumor samples (67%).^17^ CD73 expression in the primary tumor was associated with a trend towards higher nuclear grade and numerically worse 5-year OS. Our current analysis included tumor samples from 107 patients with localized RCC and 31 patients with de novo mRCC from three high-volume kidney cancer centers with well-annotated long-term clinical outcomes (median ~10 years from nephrectomy). Confirming the results of our prior study, tumors from patients with de novo mRCC had a significantly higher propensity to express CD73 (55%) compared with localized disease (23%, p=0.002). The relatively higher prevalence of CD73 expression demonstrated by Yu *et al* might be due to different antibodies used for IHC in their analysis (ab115289; Abcam, Cambridge, Massachusetts, USA). The prevalence of CD73 positivity seen in the current analysis is consistent with our prior study using the same anti-CD73 antibody (clone D7F9A, Cell Signaling Technology).

CD73 expression also correlated with adverse pathologic features and was independently associated with worse prognosis after adjusting for other prognostic features such as tumor grade and AJCC stage in patients with localized RCC. Similarly, in the TCGA dataset, high *NT5E* expression was associated with worse outcomes in patients with AJCC stage IV RCC. These findings suggest that CD73–adenosine signaling may be a potential driver of aggressive disease in patients with both localized and advanced RCC. As several agents targeting this pathway are in early clinical development, these findings provide compelling rationale for targeting this pathway not only in mRCC but also as perioperative therapy in patients with localized disease and high CD73 expression.

We also characterized CD73 expression in non-clear cell subtypes (papillary n=16, chromophobe n=14) and tumors with a sarcomatoid component (n=10), RCC disease states where expression of inhibitory immune checkpoints has been shown to play a significant immunosuppressive role.^23–26^ Approximately 5%–30% of non-ccRCCs express PD-L1, and PD-1/-L1 blockade has demonstrated encouraging efficacy in non-clear cell histologies with an ORR of approximately 25%.^23 24^ Similarly, the presence of sarcomatoid differentiation has been associated with increased responsiveness to immune checkpoint inhibition.^25 26^ The CD73–adenosine pathway could be a complementary therapeutic target in these histological subtypes where there exists an even greater clinical need for more effective therapies. We observed CD73 positivity in 39% of our non-ccRCC samples with 24% demonstrating high CD73 expression. In our dataset, 90% of tumors with a sarcomatoid component demonstrated CD73 expression, and 50% were CD73_high_. Although limited by the small sample sizes and in need of validation, to our knowledge, this is the first study characterizing CD73 expression in non-clear cell and sarcomatoid RCC.

In the TCGA dataset, we also found a strong correlation between CD73 (*NT5E*), CD39 (*ENTPD1*) and A2AR (*ADORA2A*) expression, and Treg gene expression signature. CD73 and CD39 activation on tumor and stromal cells generates extracellular adenosine, which exerts an immunosuppressive effect antagonistic to PD-1 inhibitors. Adenosine activates the high-affinity A2AR receptor, which in turn inhibits infiltrating NK cells and cytotoxic T lymphocyte activity and increases Treg proliferation.^11 27–30^ In addition to tumor cells, CD73, CD39, and A2AR can also be expressed on infiltrating immune cells such as Tregs and effector T cells with resultant autocrine production of adenosine further promoting immunosuppression through Treg proliferation.^27 31^ The strong correlation of *NT5E*, *ENTPD1*, and *ADORA2A* expression with the Treg signature observed in our analysis supports the findings of prior preclinical studies^11^ and provides additional mechanistic support for the immunosuppressive role of CD73–adenosine signaling in RCC.

Adenosine signaling also mediates the recruitment and proliferation of granulocytic MDSCs.^12^ Fong *et al* reported that a gene expression signature incorporating genes encoding CXCR2 ligands (eg, CXCL1, CXCL2, CXCL3, CXCL5, and CXCL6) and mediators of neutrophil and MDSC biology (ILB, IL1B, and PTGS2) could be a potential biomarker of CD73–adenosine signaling.^32^ The myeloid-inflammation gene signature used in our study includes several genes included in the adenosine signature (*CXCL1*, *CXCL2*, *CXCL3*, *CXCL8*, and *PTGS2*). However, we did not find a correlation between *NT5E* expression and myeloid inflammation gene expression signature. Further, there was no significant difference in MDSC signature between low and high *NT5E* expression groups (p=3.50E-01). It is possible that CD73 protein expression or cell type-specific expression (tumor vs immune cell) might have differential effects on these signatures, which was not captured in the bulk RNA-seq data derived from the TCGA dataset.

Preclinical evidence also suggests that CD73–adenosine signaling may promote angiogenesis through vasodilation, release of proangiogenic factors such as VEGF, and recruitment of endothelial progenitor cells.^14–16^ While we did not observe a correlation between *NT5E* expression and angiogenesis marker genes, high expression of *ADORA2A* was strongly associated with high angiogenesis gene expression signature. Dysregulation of the proangiogenic HIF pathway is a critical oncogenic driver in RCC, and A2AR has been identified as a downstream proangiogenic target unique to HIF-2α.^33 34^ A2AR activation can result in increased angiogenesis through decreased secretion of thrombospondin-1 (TSP-1) and by promoting differentiation of macrophages to the M2 phenotype resulting in increased expression of proangiogenic factors such as VEGF, IL-10 and nitric oxide synthase.^35–37^ Our findings, in corroboration with mechanistic insights from preclinical studies,^11 28 29 34 38 39^ support the hypothesis that adenosine signaling contributes to both tumor immune evasion and angiogenesis in RCC.

Several agents targeting the CD73–adenosine pathway are in clinical development either as monotherapy or in combination with approved anti-PD-1/L1 agents across several cancers, including mRCC. In a first in human, phase I dose-escalation study, the oral competitive A2AR inhibitor ciforadenant (CPI-444) was evaluated as monotherapy and in combination with atezolizumab in patients with advanced refractory cancers.^32^ Among patients with treatment-refractory mRCC, ciforadenant demonstrated encouraging efficacy, and disease control lasting at least 6 months was seen in 39% of patients treated with the combination. Multiple ongoing trials are investigating adenosine pathway blockade via inhibition of CD73, A2AR, and CD39 as monotherapy and combinations (eg, NCT04148937, NCT03549000, NCT03454451, NCT03835949, NCT03884556, NCT03381274, and NCT04336098). Evaluation of predictive biomarkers such as CD73 expression or adenosine pathway gene expression signatures has the potential to optimize patient selection.

Our study has limitations inherent to institutional retrospective analyses. The relatively small number of patients with non-clear cell histology and de novo mRCC limited our investigation of the prognostic significance of CD73 expression in these subgroups. CD73 expression on stromal cells or infiltrating immune cells was not assessed, which could provide additional insights into the role of CD73–adenosine signaling in the tumor microenvironment. Although our analysis demonstrates that CD73 expression is associated with poor prognosis at both the mRNA and protein level, additional experimentation will be required to determine the correlation between CD73 protein and mRNA expression. Lastly, protein expression of A2AR and CD39 was not assessed in the current dataset but is worthy of future study of optimal biomarker signatures for adenosine signaling in RCC.

## Conclusions

The CD73–adenosine pathway is an emerging therapeutic target in RCC with extensive preclinical data and growing clinical evidence supporting its role in immunosuppression and angiogenesis. We found that a significant proportion of both clear cell and non-ccRCC tumors express CD73 with a propensity for higher expression in de novo mRCC and sarcomatoid tumors. Higher expression correlated with worse DFS and OS in patients with localized disease independent of stage and grade. In addition, gene expression of CD73, CD39 and A2AR was associated with increased immunosuppressive cell markers and while A2AR expression correlated with the angiogenesis signature in the TCGA cohort. Our findings support the growing investigation of this pathway in advanced RCC.
