# Supplementary material for: Prognostic significance and immune correlates of CD73 expression in renal cell carcinoma
Source: J Immunother Cancer. 2020 Nov 11;8(2):e001467. doi: 10.1136/jitc-2020-001467 (PMC7661372; doi:10.1136/jitc-2020-001467)

SUPPLEMENTARY DATA

Supplementary Table 1: Comparison of gene expression signatures between Low, Intermediate (Int) and High *NT5E* expression groups

Gene Expression Signature	P Low vs. Int	P Int vs. High	P Low vs. High	FDR-p Low vs. Int	FDR-p Int vs. High	FDR-p Low vs. High	Reject
**Angiogenesis**	4.55E-07	6.82E-03	5.49E-04	1.37E-05	1.57E-01	1.20E-02	FALSE
**Myeloid Inflammation**	4.82E-02	3.28E-01	1.10E-01	4.99E-01	8.79E-01	3.73E-01	FALSE
**T effector**	6.71E-05	4.84E-02	9.66E-03	1.68E-03	6.15E-01	1.19E-01	FALSE
**Activated B cell**	4.88E-02	3.43E-01	4.17E-02	4.99E-01	8.79E-01	3.37E-01	FALSE
**Activated CD4 T cell**	5.23E-04	3.26E-01	5.78E-04	1.14E-02	8.79E-01	1.21E-02	FALSE
**Activated CD8 T cell**	1.83E-01	1.10E-01	2.92E-02	7.56E-01	8.05E-01	3.00E-01	FALSE
**Activated dendritic cell**	1.40E-05	1.90E-01	1.85E-03	3.79E-04	8.79E-01	2.92E-02	FALSE
**CD56bright natural killer cell**	1.13E-04	2.01E-01	6.14E-05	2.71E-03	8.79E-01	1.66E-03	FALSE
**CD56dim natural killer cell**	3.44E-07	5.05E-02	1.68E-04	1.07E-05	6.15E-01	4.02E-03	FALSE
**Central memory CD4 T cell**	1.62E-01	2.41E-02	8.12E-03	7.56E-01	4.25E-01	1.08E-01	FALSE
**Central memory CD8 T cell**	4.18E-01	2.31E-03	4.38E-02	8.03E-01	5.85E-02	3.37E-01	FALSE
**Effector memory CD4 T cell**	1.44E-02	1.13E-02	9.70E-05	2.07E-01	2.38E-01	2.42E-03	FALSE
**Effector memory CD8 T cell**	2.23E-05	1.18E-01	6.87E-04	5.79E-04	8.05E-01	1.37E-02	FALSE
**Eosinophil**	1.81E-02	6.81E-02	7.49E-04	2.39E-01	6.98E-01	1.41E-02	FALSE
**Gamma delta T cell**	2.48E-01	4.66E-02	3.23E-01	7.59E-01	6.15E-01	5.02E-01	FALSE
**Immature B cell**	4.93E-02	3.72E-02	2.59E-03	4.99E-01	5.49E-01	3.82E-02	FALSE
**Immature dendritic cell**	1.88E-04	4.46E-01	1.40E-03	4.32E-03	8.79E-01	2.35E-02	FALSE
**MDSC**	4.71E-01	2.38E-02	6.79E-02	8.03E-01	4.25E-01	3.50E-01	FALSE
**Macrophage**	1.05E-01	2.54E-01	6.80E-02	6.72E-01	8.79E-01	3.50E-01	FALSE
**Mast cell**	1.89E-01	4.36E-01	2.94E-01	7.56E-01	8.79E-01	5.02E-01	FALSE
**Memory B cell**	1.13E-03	2.27E-01	1.84E-04	2.34E-02	8.79E-01	4.22E-03	FALSE
**Monocyte**	1.13E-03	1.56E-01	3.67E-02	2.34E-02	8.69E-01	3.37E-01	FALSE
**Natural killer T cell**	2.97E-01	7.28E-02	5.97E-02	7.59E-01	6.98E-01	3.50E-01	FALSE
**Natural killer cell**	6.06E-02	7.35E-04	6.54E-05	4.99E-01	1.97E-02	1.70E-03	FALSE
**Neutrophil**	5.82E-03	2.12E-01	1.31E-03	1.05E-01	8.79E-01	2.33E-02	FALSE
**Plasmacytoid dendritic cell**	4.52E-01	6.89E-02	1.83E-01	8.03E-01	6.98E-01	4.55E-01	FALSE
**Regulatory T cell**	2.79E-06	1.92E-05	3.90E-12	8.08E-05	5.56E-04	1.21E-10	TRUE
**T follicular helper cell**	1.03E-05	1.75E-05	3.92E-11	2.88E-04	5.25E-04	1.18E-09	TRUE
**Type 1 T helper cell**	1.39E-01	1.59E-01	4.03E-02	7.40E-01	8.69E-01	3.37E-01	FALSE
**Type 17 T helper cell**	6.06E-03	6.87E-04	2.40E-06	1.05E-01	1.91E-02	6.73E-05	FALSE
**Type 2 T helper cell**	6.02E-03	5.03E-08	1.18E-08	1.05E-01	1.56E-06	3.41E-07	FALSE

Supplementary Table 2: Comparison of gene expression signatures between Low, Intermediate (Int) and High *ADORA2A* gene expression groups

Gene Expression Signature	p Low vs. Int	p Int vs. High	p Low vs. High	FDR-p Low vs. Int	FDR-p Int vs. High	FDR-p Low vs. High	Reject
**Angiogenesis**	7.83E-28	6.98E-06	4.37E-36	0.00E+00	1.33E-04	0.00E+00	TRUE
**Myeloid Inflammation**	3.91E-01	4.59E-01	3.69E-01	8.36E-01	8.60E-01	8.82E-01	FALSE
**T effector**	5.40E-18	1.93E-02	1.48E-21	0.00E+00	2.24E-01	0.00E+00	FALSE
**Activated B cell**	8.22E-07	3.66E-02	3.97E-04	1.48E-05	3.36E-01	5.86E-03	FALSE
**Activated CD4 T cell**	1.22E-12	3.93E-01	1.26E-11	3.18E-11	8.60E-01	2.77E-10	FALSE
**Activated CD8 T cell**	5.30E-07	3.25E-01	3.40E-07	1.06E-05	8.60E-01	6.81E-06	FALSE
**Activated dendritic cell**	6.40E-02	4.80E-14	8.11E-16	3.28E-01	1.44E-12	2.02E-14	FALSE
**CD56bright natural killer cell**	4.47E-01	5.42E-07	2.70E-05	8.36E-01	1.08E-05	4.87E-04	FALSE
**CD56dim natural killer cell**	6.24E-21	4.77E-02	2.32E-22	0.00E+00	3.87E-01	0.00E+00	FALSE
**Central memory CD4 T cell**	5.05E-12	4.36E-03	7.56E-09	1.26E-10	6.76E-02	1.59E-07	FALSE
**Central memory CD8 T cell**	1.56E-04	1.33E-09	8.11E-02	2.02E-03	3.60E-08	5.33E-01	FALSE
**Effector memory CD4 T cell**	2.21E-03	1.34E-07	1.37E-01	1.76E-02	2.95E-06	6.93E-01	FALSE
**Effector memory CD8 T cell**	3.60E-18	3.63E-01	1.38E-18	0.00E+00	8.60E-01	0.00E+00	FALSE
**Eosinophil**	1.96E-01	9.85E-02	4.35E-02	6.63E-01	5.64E-01	3.59E-01	FALSE
**Gamma delta T cell**	4.86E-09	6.56E-02	1.91E-06	1.07E-07	4.57E-01	3.63E-05	FALSE
**Immature B cell**	1.42E-02	9.85E-02	2.53E-03	9.54E-02	5.64E-01	2.74E-02	FALSE
**Immature dendritic cell**	2.12E-22	5.23E-15	9.97E-38	0.00E+00	1.62E-13	0.00E+00	TRUE
**MDSC**	3.04E-05	4.13E-07	4.98E-01	4.56E-04	8.67E-06	8.82E-01	FALSE
**Macrophage**	2.51E-06	2.38E-02	4.48E-04	4.26E-05	2.51E-01	5.86E-03	FALSE
**Mast cell**	5.35E-04	4.43E-08	3.51E-01	5.87E-03	1.11E-06	8.82E-01	FALSE
**Memory B cell**	4.01E-01	3.51E-01	4.08E-01	8.36E-01	8.60E-01	8.82E-01	FALSE
**Monocyte**	7.59E-04	1.80E-10	1.96E-14	6.81E-03	5.21E-09	4.91E-13	TRUE
**Natural killer T cell**	3.64E-01	7.41E-08	3.92E-04	8.36E-01	1.78E-06	5.86E-03	FALSE
**Natural killer cell**	3.78E-05	2.14E-09	3.48E-01	5.29E-04	5.57E-08	8.82E-01	FALSE
**Neutrophil**	5.70E-09	1.32E-02	1.42E-12	1.20E-07	1.81E-01	3.40E-11	FALSE
**Plasmacytoid dendritic cell**	5.87E-07	4.92E-04	7.50E-04	1.12E-05	8.33E-03	8.96E-03	TRUE
**Regulatory T cell**	1.54E-09	1.04E-04	3.37E-05	3.71E-08	1.87E-03	5.73E-04	TRUE
**T follicular helper cell**	1.75E-05	8.36E-08	2.51E-01	2.80E-04	1.92E-06	8.24E-01	FALSE
**Type 1 T helper cell**	5.41E-04	3.09E-10	1.39E-01	5.87E-03	8.65E-09	6.93E-01	FALSE
**Type 17 T helper cell**	2.65E-09	1.57E-02	4.11E-12	6.09E-08	1.99E-01	9.45E-11	FALSE
**Type 2 T helper cell**	1.94E-04	1.29E-01	6.21E-05	2.33E-03	5.64E-01	9.94E-04	FALSE

Supplementary Table 2: Comparison of gene expression signatures between Low, Intermediate (Int) and High *ENTPD1* gene expression groups

Gene Expression Signature	p Low vs. Int	p Int vs. High	P Low vs High	FDR-p Low vs. Int	FDR-p Int vs. High	FDR-p Low vs. High	Reject
**Angiogenesis**	4.18325E-24	0.415835873	1.33113E-23	0	0.957489691	0	FALSE
**Myeloid Inflammation**	0.272382913	0.365879288	0.171828358	0.480005731	0.957489691	0.368136465	FALSE
**T effector**	4.39035E-30	0.384326098	1.87486E-28	0	0.957489691	0	FALSE
**Activated B cell**	4.74908E-10	0.098600287	3.47068E-12	9.02325E-09	0.82876361	6.59427E-11	FALSE
**Activated CD4 T cell**	1.67278E-15	0.078489271	2.12014E-18	3.9968E-14	0.770381368	0	FALSE
**Activated CD8 T cell**	8.15037E-11	0.125925059	4.1784E-08	1.71158E-09	0.883914303	5.43192E-07	FALSE
**Activated dendritic cell**	0.009662161	0.368980664	0.033404112	0.083673264	0.957489691	0.156228766	FALSE
**CD56bright natural killer cell**	0.063735802	0.023165957	0.001011851	0.326417957	0.37422857	0.007061494	FALSE
**CD56dim natural killer cell**	6.72871E-27	0.248620144	8.29933E-27	0	0.956902208	0	FALSE
**Central memory CD4 T cell**	2.62163E-15	3.62573E-06	2.12242E-21	6.12843E-14	0.000101515	0	TRUE
**Central memory CD8 T cell**	0.00027132	0.215097045	0.000166459	0.002980472	0.947507965	0.001663346	FALSE
**Effector memory CD4 T cell**	6.80351E-07	0.012277749	1.65413E-10	1.08856E-05	0.237979912	2.84834E-09	FALSE
**Effector memory CD8 T cell**	5.10928E-33	0.358304253	7.89604E-33	0	0.957489691	0	FALSE
**Eosinophil**	0.002423853	0.251951865	0.000436363	0.023975855	0.956902208	0.003485576	FALSE
**Gamma delta T cell**	1.41025E-06	0.004316079	2.03176E-08	1.83331E-05	0.098603536	2.84446E-07	FALSE
**Immature B cell**	0.094587763	0.140570705	0.012258841	0.391540329	0.896925249	0.071335368	FALSE
**Immature dendritic cell**	5.68914E-30	0.000349844	6.38711E-34	0	0.009056287	0	TRUE
**MDSC**	9.55839E-07	0.031239847	3.20506E-09	1.33817E-05	0.452846632	4.80759E-08	FALSE
**Macrophage**	1.20912E-10	0.288817677	1.58241E-10	2.41825E-09	0.956902208	2.84834E-09	FALSE
**Mast cell**	0.179171615	0.391781508	0.314610278	0.480005731	0.957489691	0.368136465	FALSE
**Memory B cell**	0.01015126	0.202839727	0.089276934	0.083673264	0.947507965	0.312068265	FALSE
**Monocyte**	7.14201E-06	0.39638948	9.61746E-05	8.57007E-05	0.957489691	0.001057412	FALSE
**Natural killer T cell**	0.046665988	0.008904332	0.000324513	0.284325383	0.185936491	0.002916827	FALSE
**Natural killer cell**	8.61035E-07	5.65779E-08	1.49486E-17	1.29154E-05	1.64076E-06	0	TRUE
**Neutrophil**	2.63899E-14	0.000223255	1.81924E-20	5.81313E-13	0.006010424	0	TRUE
**Plasmacytoid dendritic cell**	1.32509E-09	0.149091769	1.56457E-09	2.25266E-08	0.896925249	2.50331E-08	FALSE
**Regulatory T cell**	2.51999E-27	5.67783E-09	1.5765E-37	0	1.70335E-07	0	TRUE
**T follicular helper cell**	5.27695E-10	0.014759917	4.59707E-13	9.4985E-09	0.268214983	9.19487E-12	FALSE
**Type 1 T helper cell**	0.2099327	0.32615225	0.141893686	0.480005731	0.957489691	0.368136465	FALSE
**Type 17 T helper cell**	1.11412E-17	0.002968156	3.19536E-22	0	0.07162008	0	FALSE
**Type 2 T helper cell**	0.15082023	3.65469E-14	4.4812E-06	0.480005731	1.13232E-12	5.3773E-05	FALSE

Supplementary Figure 1: Patient selection for the DF/HCC institutional dataset

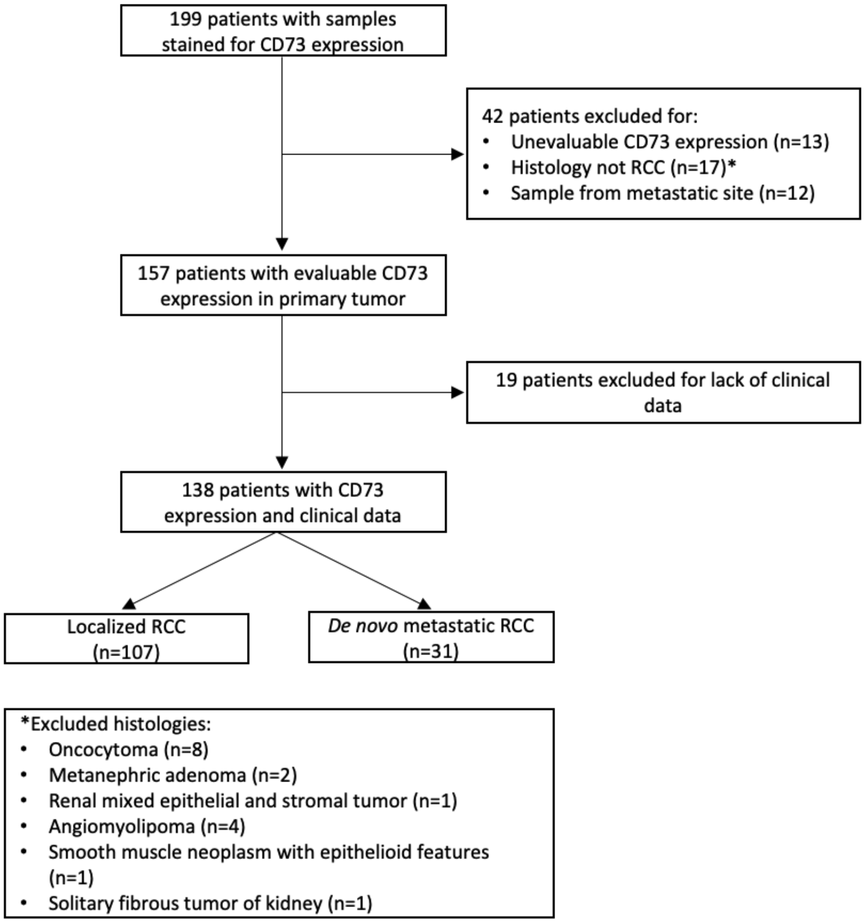

Supplementary Figure 2: Kaplan-Meier plot of OS according to CD73 expression in patients with *de novo* metastatic RCC

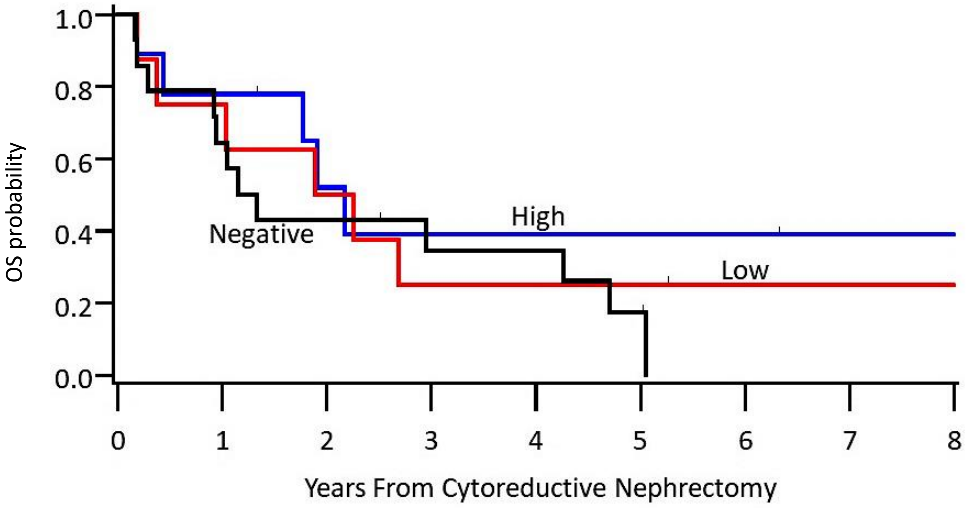

Negative	14	6	4	0	0
Low	8	4	2	1	1
High	9	4	3	3	2

Supplementary Figure 3. Correlation of *NT5E* expression in TCGA cohort with overall survival (A, B and C) and AJCC stage (D)

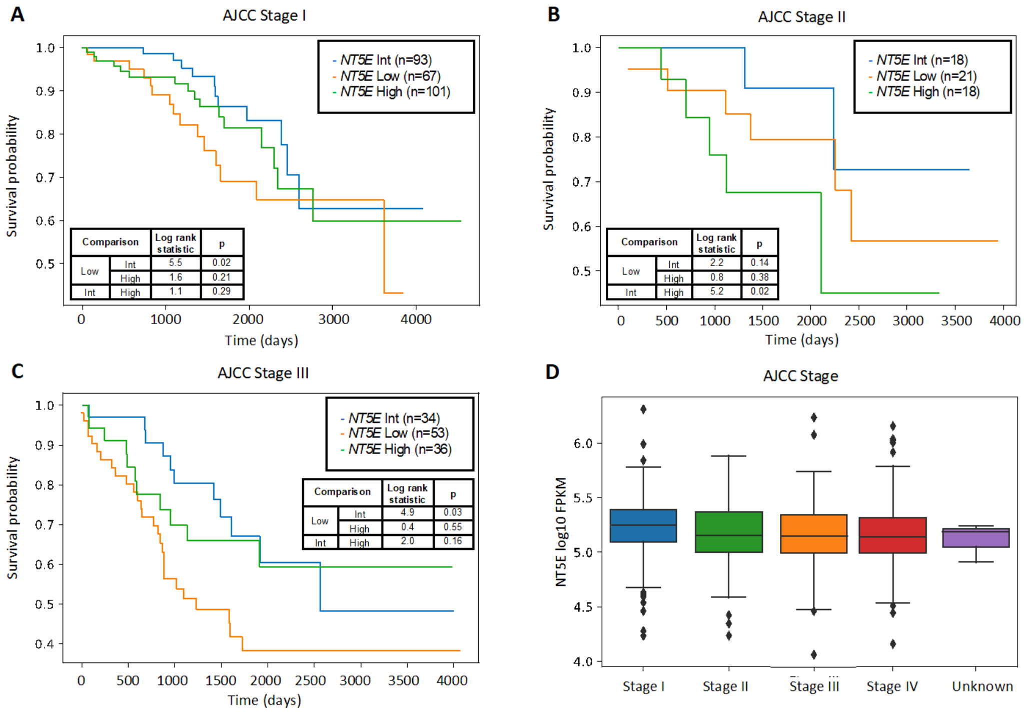

Supplementary Figure 4: Correlation of *ADORA2A* expression in TCGA cohort with overall survival (A, B , C and D) and AJCC stage (E)

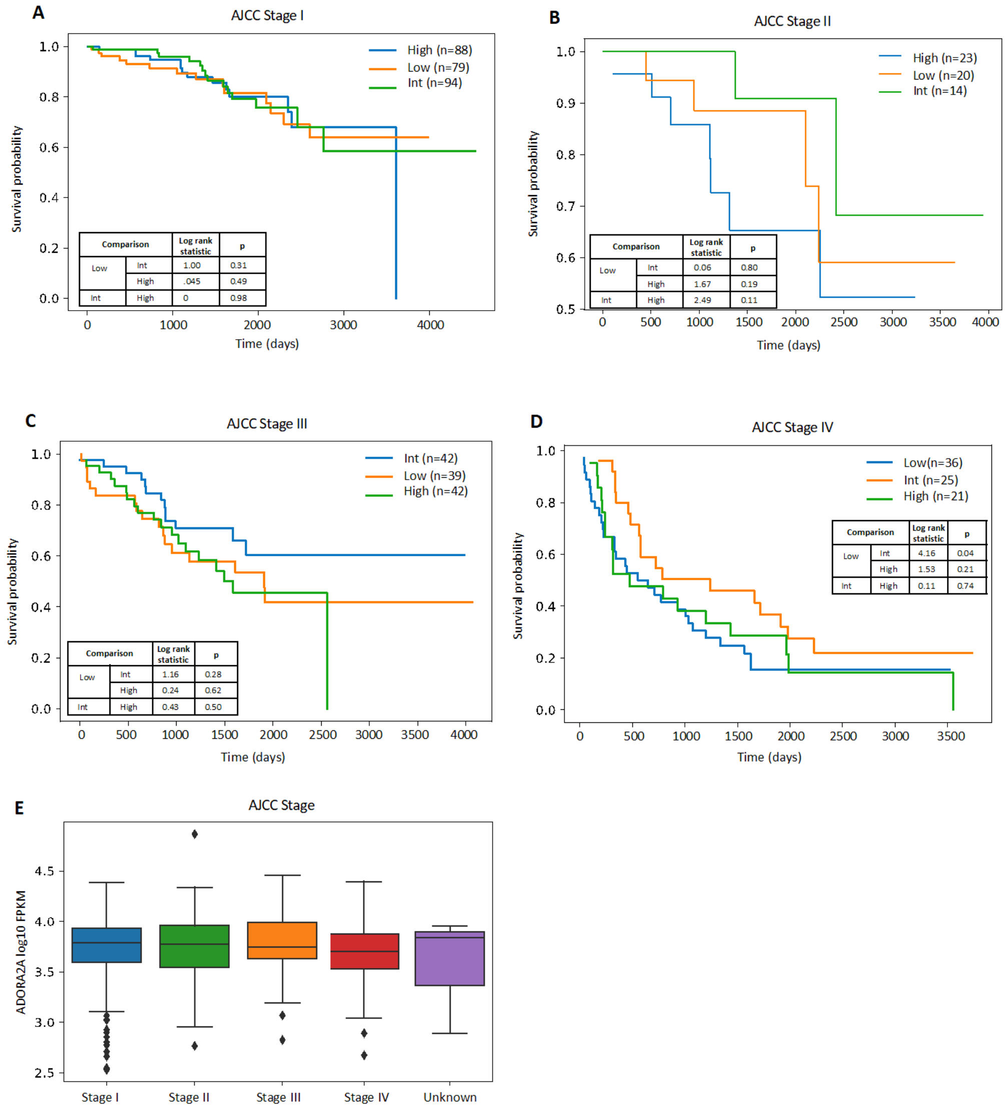

Supplement: Supplementary data [file jitc-2020-001467supp001.pdf]
